# Veterans Affairs Health Care Provider Perceptions of Virtual Reality: Brief Exploratory Survey

**DOI:** 10.2196/38490

**Published:** 2022-09-02

**Authors:** A'mie M Preston, Lana Brown, Kalpana P Padala, Prasad R Padala

**Affiliations:** 1 Geriatric Research Education and Clinical Center Eugene J Towbin Veterans Affairs Healthcare Center North Little Rock, AR United States; 2 College of Nursing University of Arkansas for Medical Sciences Little Rock, AR United States; 3 Department of Geriatrics University of Arkansas for Medical Sciences Little Rock, AR United States; 4 Department of Psychiatry University of Arkansas for Medical Sciences Little Rock, AR United States; 5 University of Arkansas for Medical Sciences Graduate Medical Education Baptist Health North Little Rock, AR United States

**Keywords:** virtual reality, older adults, provider perception

## Abstract

**Background:**

Virtual reality (VR), a simulated experience that can be similar to or completely different from the real world, has become increasingly useful within the psychiatric and medical fields. This VR technology has been applied in medical school trainings, exposure therapy for individuals with posttraumatic stress disorder (PTSD), and reminiscence therapy associated with mood disorders for older adults. Perceptions of VR through the lens of the health care provider require further exploration. VR has grown in popularity; however, this modality continues to be underused in most Veterans Affairs (VA) hospitals.

**Objective:**

A web-based survey was used to explore health care provider perceptions of immersive VR availability and use for older adults and identify potential barriers for immersive VR use in older adults with cognitive impairment.

**Methods:**

An 8-item web-based survey was developed to obtain health care provider feedback. This survey was disseminated throughout a single Veterans Integrated Services Network (VISN). The VR survey was developed via the Survey Monkey platform and distributed through the secure VA email network. Providers were asked to voluntarily participate in the brief, anonymous survey and offer their perceptions of immersive VR use within their patient population. Survey data were reviewed and interpreted using descriptive statistics.

**Results:**

A total of 49 respondents completed the survey over a 15-day period. Of them, 36 respondents (73%) had heard of a VR device, though the majority (n=44, 90%) had never used or prescribed a VR device. Respondents identified several potential barriers to immersive VR use in older adults with cognitive impairment (eg, hearing difficulties, perceptions of technology, cognitive concerns, access to resources, and visual impairment). Despite the barriers identified, providers (n=48, 98%) still reported that they would feel comfortable prescribing immersive VR as an intervention for their patient population.

**Conclusions:**

Survey findings revealed that health care providers within this VISN for VAs have heard of VR, although they may not have actively engaged in its use. Most of the providers reported that they would prescribe the use of an immersive VR intervention for their older adult patients. This key point highlights the desire to implement VR strategies for patient use by their providers. If underlying barriers can be addressed and relatively resolved, this technological intervention has the potential to create substantial breakthroughs in clinical care.

## Introduction

Nonpharmacological interventions in health care are an alternative approach to pharmacological therapies and are recommended as a first-line treatment prior to introducing medications. This is especially important for older adults due to the increased risk for adverse outcomes. One nonpharmacological intervention with increasing recognition and application among health care providers involves the use of virtual reality (VR) devices. For the purposes of this survey, health care providers include any licensed independent provider or clinician who provides health care to older adults (eg, physicians, nurses, and psychologists). These VR devices use technology to create a simulated environment. VR is a computer-generated simulation in which one can interact with the surrounding environment by using various devices such as computers, smartphones, or head-mounted devices that can be immersive or nonimmersive [1]. In this survey, immersive VR was described to participants and used as the type of VR experience. VR has been shown to be a viable option for older adults [2]. Its design allows for a more immersive experience by reducing external distractions [3]. It is rapidly expanding as an intervention for many diagnoses and clinical presentations. In mental health, VR abilities provide its users with a virtual exposure (vs in vivo) to stimuli, making it an ideal intervention for addressing posttraumatic stress disorder (PTSD) [4]. VR has been used to treat mental health diagnoses of anxiety [5], eating disorders [6], obesity [7], sexual dysfunctions [8], phobias [9], and PTSD [10,11].

The medical education field has also used VR to educate providers on various medical conditions [12,13], have a cost-effective way to teach students in medical school programs [14], and training them in team building exercises [15]. Provider perception is key to implementing appropriate interventions and referral options for their patients. Limited research exists in the area of health care providers prescribing VR use for their patients. The hesitancy of psychotherapists to prescribe VR devices has been explored in research [16]; however, hesitancy from other health care providers has yet to receive as much attention.

Ways to seamlessly integrate the use of technology in older adults has been a topic of interest since the late 1980s [17]. More recently, VR use has been studied in cognitively impaired older adult population for neuropsychiatric testing [18,19], cognitive rehabilitation [20], and reducing neuropsychiatric symptoms associated with dementia [21]. Researchers have worked to find innovative ways to reduce neuropsychiatric symptoms in those diagnosed with dementia. An additional way VR has been used in older adults is through exploring its use in reducing symptoms of apathy [22]. In studies such as these, VR was used by engaging the individual in experiences that involved music, art, cognitive stimulation, and reminiscence therapy [23]. Other uses have included memory enhancement training in those with a previous diagnosis of dementia [24]. Additionally, the use of VR could reduce medication burden in the medical field for varying mental health conditions. Increasing nonpharmacological techniques for older adults has the potential to aid the individuals themselves and their families and reduce the national financial burden of caring for these individuals. In order to connect these individuals to nonpharmacological approaches, such as VR, it is important to understand how their treating providers view VR and explore possible barriers for providers prescribing its use to their older adult population.

Although there is strong evidence to support the use of VR in older adults, it is important to understand if health care providers support the use of immersive VR in their patient populations. This survey was developed as preparatory to a funded research study to examine the use of immersive VR in older veterans with cognitive impairment. The aim was to use a web-based survey to explore health care provider perceptions of immersive VR availability and use in older adults as well as to identify potential barriers for VR use in older adults with cognitive impairment. Through analysis of the responses, possible obstacles or stigma were identified to better understand current perceptions and advise future directions for immersive VR use with older adults.

## Methods

### Participants

All health care providers from a single Veterans Affairs (VA) Veterans Integrated Services Network (VISN) were invited to participate in a web-based, anonymous, closed survey. Health care providers were selected from one VA VISN that included 8 VA medical centers to better understand views from this specific population. The Veterans Affairs Health Administration is divided into 18 VISNs (or regional systems) across the nation. These regional divisions are meant to increase access to care and resources for veterans throughout the United States.

### Survey Design

The survey, included in Multimedia Appendix 1, was developed by an experienced nurse researcher with vast knowledge of education and evaluation and an experienced psychology researcher working on immersive VR research. Participants were allowed to take the survey only once, based on the computer IP address. Author expertise in this area includes prior experience with implementing VR in a clinical setting and as part of a national research study. Through combined experiences, the authors were aware of a portion of the potential obstacles and benefits related to provider buy-in with immersive VR devices. A more focused look into this area is needed to aid in further exploration of the field. The web-based survey was developed on the Survey Monkey platform and emailed out to the health care providers within one VA VISN for voluntary feedback. A brief explanation of immersive VR and the purpose of the survey were included in the email with the link to the survey. The estimated time of survey completion was also included. The 8-item survey was made available for approximately two weeks for participants to complete. No incentives were offered to participants for completing the survey.

### Ethical Considerations

The Institutional Review Board of the Central Arkansas Veterans Health Care System deemed the survey as nonresearch and preparatory to research activity and therefore exempted it from ethics review.

### Statistical Analysis

Descriptive statistics were used to analyze, summarize, and present the survey data findings. Frequency distribution was used to depict the frequency or count of different outcomes in the sample. The frequency distribution was presented in a table format to summarize the health care disciplines and potential VR barriers in older adults with cognitive impairment. Each entry in the table was accompanied by the count or frequency along with associated percentages.

## Results

A total of 49 participants completed the survey. Table 1 displays disciplines for the participants of the project. Each participant who self-identified as a physician was asked to specify their discipline. Of the physicians who completed the survey, their disciplines were identified as follows: 5 psychiatry, 1 geriatrician, 2 family medicine, 1 ophthalmology, 1 primary care or internal medicine, and 1 behavioral health. Of note, one participant who identified as an administrator noted being a physician of family medicine. This participant was not included in the overall physician count. Of 49 participants, 36 (73%) reported that they had heard of VR devices. A substantially smaller portion (n=5, 10%) reported previously using the device or prescribing the device for a patient. Of the individuals who reported previously using or prescribing the VR device, 12 (25%) reported that either themselves or their patients did not enjoy the experience. When asked if providers believed that immersive VR devices could be used with older adults, 46 (94%) reported yes. A distinction was made between using immersive VR with older adults with cognitive impairment and those without it. When asked if immersive VR could possibly be used with older adults with cognitive impairment, most providers (n=42, 86%) still reported yes. Overall, an overwhelming majority of providers (n=48, 98%) reported that they would refer their patients to a program that uses immersive VR devices. Respondents were given an option to check certain barriers that they felt may be present for older adults when using immersive VR devices or write in barriers that were not included on the preestablished barrier list. Table 2 depicts the breakdown of barriers identified by the respondents. From the participants who marked “other” for identified barriers, the following were reported as possible barriers to immersive VR use: mental health issues (eg, PTSD), connectivity issues in rural areas, resistance to device going over their head (especially in older adults with dementia), apathy, resistance to new technology, and unwillingness to make changes.

**Table 1:** Percentage of health care providers by discipline (N=49).

Provider disciplines	Values, n (%)
Social worker	14 (29)
Nursing	10 (21)
Physician	10 (20)
Psychologist	6 (12)
Administration	3 (6)
Chaplain	2 (4)
Physical therapist	1 (2)
Recreation therapist	1 (2)
Neuropsychologist	1 (2)
Research health specialist	1 (2)

**Table 2:** Perceived barriers to virtual reality use by health care providers (N=12).

Barriers	Values, n (%)
Cognitive issues	36 (74)
Perceptions of technology	36 (74)
Hearing loss	35 (71)
Vision problems	33 (67)
Access to resources	28 (57)
Frailty	10 (20)
Mood issues	8 (16)
Other	5 (10)

## Discussion

### Principal Results

Our data yielded results highlighting that health care providers agreed that VR devices can be used with older adults, and furthermore, with older adults who have cognitive impairment. This information matches previous data in studies where older adults engaged in VR-based research [1,23,24]. Although providers highlighted barriers to immersive VR use in this population, the majority of health care providers would refer their patients to a program where immersive VR devices are used. Key barriers included physical, social, emotional, and cognitive obstacles that providers believed could hinder VR use in older adults with cognitive impairment.

### Interpretation of Results

Results of this project are consistent with literature in the field addressing provider acceptance of VR use for patients. Additionally, social barriers identified by providers regarding the use of VR devices in practice have been reported [25]. A limited number of studies have explored physical factors (eg, frailty, vision impairments, or mobility issues) displayed by patients that providers believe can inhibit the use of VR devices. These physical factors have been identified as barriers for other social groups, although they have been highlighted more frequently with older adults. The two significant barriers identified in the above-mentioned study by providers were cognitive issues displayed by the patient and patient’s perceptions of technology. Even though cognitive impairment can serve as a hinderance for engagement in some VR activities, VR has also acted as an assessment tool for diagnosing mild cognitive impairment [26]. Researchers have found that VR applications can be used toward improvement of cognitive impairment [27,28]. Few studies have thoroughly evaluated the perception of technology as viewed by older adults with cognitive impairment.

### Comparison to Prior Work

Chung et al [29] conducted a similar type of work, where perceptions of VR were explored with clinicians and service managers. Their study included barriers not only from the clinician’s view but also from the patient’s and the organization’s view. Three broad themes emerged from the analysis of the survey barrier data (clinical factors, organizational factors, and professional factors). Overall, participants of the project agreed that VR could be used to break down barriers of care. Additionally, participants noted that VR could prove to be a beneficial mental health intervention for those who may have found traditional psychotherapy methods to be ineffective [30]. Two main barriers for implementation of VR use addressed in their work include concerns for proper safety or ethics and concerns for resourcing (eg, staff, costs, or space). An additional study found that providers find VR to be a valuable tool and would continue to use it in their own practice [27].

### Limitations

One major limitation of our study is the small sample size reported; the project yielded results from 49 respondents. Second, we included only 8 items for the total survey. It is possible that additional information was unable to be analyzed due to the low item number. The third limitation includes the restriction of sending the survey to only one VA VISN and only VA health care providers. Some health care providers may have other roles in different care settings, though they would have required a VA affiliation to have access to this project survey.

### Future Work

Future areas of consideration include understanding the perception of health care providers toward using VR or referring patients to engage in immersive VR-based interventions. Similarly, research can explore perceptions of technological use, specifically immersive and nonimmersive VR use, in older adults with cognitive impairment. Identifying additional barriers for both forms of VR use and brainstorming possible solutions to these barriers are also required in the future.

### Conclusions

Immersive VR continues to emerge as a viable option for a myriad of clinical presentations and medical education purposes. Various uses of this technology are being identified and researched. Although systemic, professional, and individual barriers have been highlighted, the field continues to address these obstacles with the hopes of improving the overall VR experience.

## Appendix

Multimedia Appendix 1 Veterans Affairs health care providers perceptions of virtual reality survey.

## Abbreviations

PTSD: posttraumatic stress disorder
VA: Veterans Affairs
VISN: Veterans Integrated Services Network
VR: virtual reality

## Data Availability

The deidentified data sets generated and analyzed for this study are available from the corresponding author upon reasonable request.
