# Supplementary material for: Veterans Affairs Health Care Provider Perceptions of Virtual Reality: Brief Exploratory Survey
Source: Interact J Med Res. 2022 Sep 2;11(2):e38490. doi: 10.2196/38490 (PMC9482067; doi:10.2196/38490)
Supplement: Multimedia Appendix 1 [file ijmr_v11i2e38490_app1.docx]

VA Healthcare Providers Perceptions of Virtual Reality Survey

1. Have you heard of a virtual reality device before?

☐Yes ☐No

1. Have you prescribed or used a virtual reality device before?

☐Yes ☐No

1. If used before, did you enjoy the experience? If never used, skip to #4.

☐Yes ☐No

1. Do you think this device could be used with older adults?

☐Yes ☐No

1. Do you think this device could be used with older adults who have cognitive impairment?

☐Yes ☐No

1. Would you refer your patients to a program where they could use a virtual reality device?

☐Yes ☐No

| ☐Hearing  ☐Mood Issues | ☐Cognitive Issues  ☐Frailty | ☐Other (if checked, specify) |
| --- | --- | --- |

1. What barriers do you feel may keep older adults from using the device?

| ☐Vision | ☐Perceptions of Technology |
| --- | --- |

☐Access to Resource

1. How would you describe your background?

☐Primary Care Provider ☐Geriatrician ☐Mental Health Provider ☐APN/APRN ☐Other:
